# Dengue haemorrhagic fever: a job done via exosomes?

**DOI:** 10.1080/22221751.2019.1685913

**Published:** 2019-11-12

**Authors:** Ritu Mishra, Sneh Lata, Amjad Ali, Akhil C. Banerjea

**Affiliations:** a Laboratory of Virology, National Institute of Immunology, New Delhi, India; b Jamia Millia Islamia, Okhla, New Delhi, India

**Keywords:** Dengue virus, dengue haemorrhagic fever, microRNA, exosomes, hyperpermeability

## Abstract

Dengue fever is one of those unique diseases where host immune responses largely determine the pathogenesis and its severity. Earlier studies have established the fact that dengue virus (DENV) infection causes haemorrhagic fever and shock syndrome, but it is not directly responsible for exhibiting these clinical symptoms. It is noteworthy that clinically, vascular leakage syndrome does not develop for several days after infection despite a robust innate immune response that elicits the production of proinflammatory and proangiogenic cytokines. The onset of hyperpermeability in severe cases of dengue disease takes place around the time of defervescence and after clearance of viraemia. Extracellular vesicles are known to carry biological information (mRNA, miRNA, transcription factors) from their cells of origin and have emerged as a significant vehicle for horizontal transfer of stress signals. In dengue virus infection, the relevance of exosomes can be instrumental since the majority of the immune responses in severe dengue involve heavy secretion and circulation of pro-inflammatory cytokines and chemokines. Here, we present an updated review which will address the unique and puzzling features of hyperpermeability associated with DENV infection with a special focus on the role of secreted extracellular vesicles.

## Introduction

Dengue fever is caused by the infection of dengue virus. It is a mosquito-born disease identified just 100 years ago [1]. It has re-emerged in the past decade with an increasing geographic distribution of both the virus and its vector mosquitoes. Among other factors, alarming global climatic changes are the biggest contributors for such a rapid spread of this disease. Due to the increasing global temperature, about half of the world’s population is now at risk (WHO-2019). Dengue virus, the causative agent of dengue fever, is transmitted mainly by female *Aedes aegypti* and to a lesser extent by *A. albopictus*. Primary dengue infection and disease is manifested as mild and self-limiting illness in the majority of individuals. Dengue Haemorrhagic Fever (DHF), popularly known as “severe dengue,” was first recognized in the Philippines and Thailand during dengue epidemics. Severe dengue has now spread in most Asian and Latin American countries and poses a heavy burden on healthcare cost, hospitalization and death in both children and adults (WHO). Recent WHO reports indicate 390 million dengue infections per year although actual numbers of dengue cases are often underreported and misclassified due to the similarity of symptoms with other viral diseases; still almost 3.9 billion people, spanning around 128 countries, are at risk of dengue infection [2].

Dengue virus belongs to family flaviviridae. The *Flavivirus* genus contains more than 70 viruses which are transmitted by arthropods [3]. To date, dengue infections are predominantly believed to be caused by four antigenically distinct serotypes, i.e. Dengue Virus DENV-1, DENV-2, DENV-3, and DENV-4. These four serotypes are genetically similar and share approximately 65% of their genomes [4]. DENV-5 is the fifth and latest addition identified in October 2013 [5]. DENV-5 follows the sylvatic cycle (where humans are dead end host) unlike the rest four serotypes which follow the human cycle.

## What is dengue haemorrhagic fever?

Patients with dengue infection progress through three typical clinical phases; the febrile phase, the critical phase and the recovery phase (as per WHO guidelines, 2011). It begins with an initial incubation period of typically 3–7 days followed by a sudden onset of high fever along with high viraemia, known as the febrile phase (WHO guidelines, 2011). Some patients proceed to the critical phase which lasts for 24–48 h and is associated with plasma leakage. Interestingly some patients might revert to the recovery phase without experiencing plasma leakage [6]. In a few extreme cases, it causes severe clinical manifestations known as dengue haemorrhagic fever or Dengue Shock Syndrome (DHF/DSS). This condition is popularly termed as “Severe dengue.” In this review, we would use the term DHF for describing these severe clinical manifestations. This situation arises due to the transient increase in vascular permeability caused by endothelial dysfunctions. This is the most life-threatening effect of DENV infection which manifests as destabilization of the microvascular endothelial barrier resulting in plasma leakage, hypovolemic shock and ultimately haemorrhages. Blood capillaries are disrupted and heavy bleeding in vital organs, such as liver, spleen, intestines, happens in a significant proportion of individuals[7, 8]. Alterations in the microvascular circulation result in reduced blood flow and plasma leakage takes place during the critical phase. Currently a new term “capillary leak syndrome” has been used to describe vascular dysfunctions upon dengue infection [9].

## How dengue haemorrhagic fever manifests?

Vascular hyperpermeability and plasma leakage are two major hallmarks of DHF. A plethora of research carried out around the world has attempted to pinpoint the cause of this severe dengue. The roles of many cytokine mediators, mast cell products [10–12], inflammatory lipid mediators [13–15] and disruption of endothelial glyocalyx by dengue NS1 [16–18] leading to vascular leak have been reported. This has been broadly reviewed by Malavige *et.al*. [19]. Since the beginning of the research on DENV pathogenesis, the uncontrolled production of pro-inflammatory cytokines, also known as the “cytokine storm,” has been held responsible for vascular damage, hyperpermeability and shock. In the case of primary dengue fever, haemorrhagic outcomes are very less likely to happen. To explain this phenomenon, a theory of heterologous infection has been proposed. This theory suggests that upon secondary infection, there is sub-optimal neutralization of viral particles. This allows viral-antibody complex binding with fragment crystallizable (Fc) gamma receptors (FcγRs) resulting into more viral entry inside host cell, thereby enhancing viral replication [20]. This phenomenon is widely known as ADE (**A**ntibody-**D**ependent **E**nhancement) and it was first reported in 1970 by Halstead [21].

Here we will provide a detailed explanation for DHF and address all currently prevalent hypotheses of DHF and try to highlight their major limitations in explaining the complexities of the disease.

## ADE (Antibody-Dependent Enhancement) is a pre-requisite for haemorrhagic fever?

ADE is a very unique process, where pre-existing virus-specific antibodies help more viral entry in host cells and thereby help the virus rather than host. Other than DENV, HIV-1, Ebola, and Influenza viruses are also known to exhibit ADE. These viruses share a few common attributes such as preferential replication in macrophages/monocytes, ability of persistence and a greater antigenic diversity [22]**.**

The idea that antibodies produced during a primary DENV infection might be a prime cause of severe DENV condition (through ADE) remained controversial for a long time. However, this conclusion was based on extensive epidemiological studies. Severe dengue disease is frequently observed among first-time-infected infants born to DENV-immune mothers. Those children who had developed a mild or asymptomatic dengue previously were found highly susceptible for developing haemorrhagic fever or severe dengue when infected by a different DENV serotype [22, 23].

Many recent reports have kept this debate alive whether ADE alone is enough to explain dengue haemorrhagic fever. First, many reports of dengue haemorrhagic fever in the primary infection (although lesser extent) indicate that antibody-dependent enhancement is not an essential pre-requisite for plasma leakage [1]. Second, although in absolute terms, DHF patients show higher virus titre compared to patients of only dengue fever. But these titres are several logs below peak levels by the time vascular leakage occurs, and finally there are many case studies which reported that high virus titres in patients were not at all accompanied by plasma leakage [24]. Besides these, many convincing studies by using pre-infection serum from patients showed a highly variable result in generating antibody-dependent enhancing activity *in vitro* [25]. Thus, ADE phenomenon cannot be solely attributed for worsening of dengue severity. These findings strongly suggest that elevated viral load due to ADE alone is not the direct cause of vascular leakage and probably other mechanisms play an important role in triggering and contributing towards DHF phenomenon.

## Is DENV directly responsible for haemorrhage?

Dengue virus is neither a direct causative agent for hyperpermeability nor the acute cytokine storm could be the prime reason for hyperpermeability *in vitro,* at least in the early phase of infection [26]. In addition, most of the severe manifestations occur after viral clearance, implying that the immune response elicited and perpetuated by the virus could be a major cause of pathology. This mystery still remains unresolved that peak DENV leakage syndrome is observed when the viral titre starts declining in blood [21, 27]. It is puzzling to observe that vascular leakage syndrome does not appear for several days after infection despite a robust innate immune response displayed as production of proinflammatory and proangiogenic cytokines during the early phase of infections. Hyperpermeability starts exhibiting itself only around the time of defervescence and viral clearance [1]. In contrast to this, cytokine storm happens in many inflammatory diseases (like sepsis, avian influenza, smallpox, systemic inflammatory disease, etc.), but hyperpermeability or symptoms like DHF are not reported to be associated with these diseases [28, 29].

These varying observations indicate towards the involvement of a much more complex series of events related to hyperpermeability and suggest a lack of direct cause and effect relationship. Extremely low percentage of dengue virus-infected cells in blood and yet massive vascular leakage syndrome across the host suggests the role of some bystander/passive effect for causing this increased vascular permeability. The source/cause of such hyperpermeability could be diffusible and capable of intercellular communication without coming in direct contact with viral particle or antigens. This would also give virus a survival advantage since they would be able to escape the host immunity but still transmitting their characteristic vascular pathology in the host.

## Can “original Antigenic Sin”explain the severe dengue?

Original antigenic sin is also known as Hoskin’s effect. This phenomenon can be understood as “preferential boosting of pre-existing cross-reactive antibodies upon sequential infections” [30]. This makes the host inefficient in clearing the secondary infection, thereby worsening the disease [30, 31]. This phenomenon is observed mostly in those viral infections where virus generates complex antigenic variants because of the high rate of recombination and mutation like Influenza, HIV-1, and Dengue virus.

In the case of DENV infection, cell-mediated immunity; both CD4^+^ and CD8^+^ T cells are reported to help resolve DENV infection. Upon primary DENV infection, serotype-specific CD4^+^ and CD8^+^ T-cell responses have been observed [32]. DENV-specific human CD4^+^ T cells have been discovered predominantly against NS3, NS1, and envelope antigenic epitopes. CD4^+^ T cells specific for NS3 proliferate and usually produce IFN-γ and lyse the virus-infected cells [33]. However, a skewed and abnormal T-cell response has been observed during the secondary infections with a different DENV serotype which causes the “cytokine storm” and consequent immunopathogenesis of DHF. Studies have shown that in the case of the secondary infection, serotype cross-reactive T cells are preferentially activated and show lesser degranulation and enhanced TNF-α and IFN-γ production [34]. Serotype cross-reactive DENV-specific T-cells have been extensively reported to show higher production of pro-inflammatory cytokines. This less granulation and low lytic activity of T cells limits the elimination of infected cells rather induces immunopathology and causes higher disease severity during the secondary infections [35–37]. The presence of a pre-existing pro-inflammatory pattern in T-cells is associated with the development of severe dengue. Another study had shown that DENV-specific CD4^+^ T cells produce a higher ratio of TNF-α to IFN-γ upon stimulation with a heterologous antigen as compared to stimulation with homologous serotype [37, 38]. It is well established that increased TNF-α production by T cells has the potential to facilitate vascular leakage [39–41]. It is also frequently observed that TNF-α levels are much higher in the serum of patients with DHF than in the serum of DF patients [42]. Earlier studies have shown that patients with DHF exhibit greater T-cell activation *in vivo* than patients suffering from only DF (based on serum markers of activation) [24, 42–44]. These results strongly suggested that DENV-specific TNF-α secreted by memory T cells is a risk factor for the occurrence of severe dengue disease [33]. It also corroborates the theory that DHF is mostly associated with a secondary infection. However, the issue of haemorrhagic manifestation during the primary DENV infection still remains unresolved.

“Original antigenic sin” in DENV infections also has a protective role in addition to its role in pathogenesis [42]. In a recent report, it was shown that although usual skewing of responses towards the primary DENV infection was detected but that did not impair the T-cell immune responses either qualitatively or quantitatively[31].

## Can extracellular vesicles (EVs)/Exosomes provide the missing puzzle?

Studies report only a small percentage (2%) of endothelial cells to be productively infected *in vitro* by DENV with no significant apoptosis [45]. This prompts us to question which population of cells; monocytes/macrophages or lymphocytes could be the major cause of the extensive vascular leakage observed in dengue haemorrhagic fever. Multiple haemorrhagic lesions in patients observed across dermal surface /skin vasculature, gum bleeding, mucosal endothelial breakage compel us to consider the following points. First, the haemorrhagic factors are circulated via blood/plasma that is why almost all fine blood capillaries throughout body experience this leakage syndrome. Upon DENV infection, immune cells respond via secreting extra-ordinary amount of pro-inflammatory factor (could be microRNA/transcription factors/cytokine/chemokines) in bloodstream to trigger the hyperpermeability syndrome [46]. This strongly suggests the possibility that small microvesicles secreted by almost any healthy cells could be a potential hijack point for transporting these factors to reach every nook and corner of the host. Given the importance of recently recognized role of exosomes in transferring important biological materials (microRNA/transcription factors/cytokines/chemokines) in various diseases [47–49], it is logical to assume that these microvesicles could be instrumental in fulfilling viral needs of spread and pathogenesis.

In general, EVs have been demonstrated to exert a bidirectional regulatory effect on host–pathogen interaction. It can deliver its content to either promote the infection or suppress the infection. Pathogenic interests are promoted via (1) transmission of pathogen-related molecules like viral RNA or proteins, (2) immune escape of pathogen, and (3) dampening of the host immune responses. On the other hand, EVs are shown to negatively regulate pathogen via transmission and induction of antiviral responses in host immune cells (monocyte-macrophages, NK cells, T cells, and B cells) [50]. Current reports have established them as “bioactive vesicles” that perform the intercellular communication via transporting proteins, lipids and RNAs from cell to cell in various immunomodulatory events. Exosomes are generated intracellularly via typical process of endosomal invagination to form multivesicular bodies (MVBs). In most updated classification exosomes are divided into “small exosomes” and “large exosomes” [51]. Recently, another previously unappreciated nanoparticles “exomeres” have been identified [51]. Exomere is a small nanoparticle of almost 35 nm in diameter and it was reported to be carrying rather distinct protein, lipid, RNA and DNA profile as compared to normal exosomes [52]. Ordinarily, exosomes measure between 50 and 100 nm; possess a density between 1.13 and 1.19 g/ml in sucrose density gradients and exhibit a cup-shaped morphology when viewed under transmission electron microscopy [53].

All kind of immune cells, like DCs, T cells, B cells, mast cells, monocytes, and macrophages, are engaged at some time point in dengue virus life cycle and cross-talk between them seems to happen in a very dynamic and temporal fashion. Exosomes released by these cells suggest the participation of these microparticles as a preferred choice of transportation. Exosomes are even shown to contribute towards an optimal T-cell response rather than direct antigen presentation by macrophages and DCs [50]. Like other viruses, Dengue virus also changes the composition of exosomes released from infected cells [54]. A full-length dengue virus genome and several proteins have been detected in the exosomes released from infected arthropod cells [55]. The size of exosomes released from DENV-infected cells were also found to be more than those exosomes secreted from uninfected healthy cells [56]. This is probably to accommodate the whole viral genome and proteins inside exosomes.

**Figure 1.**
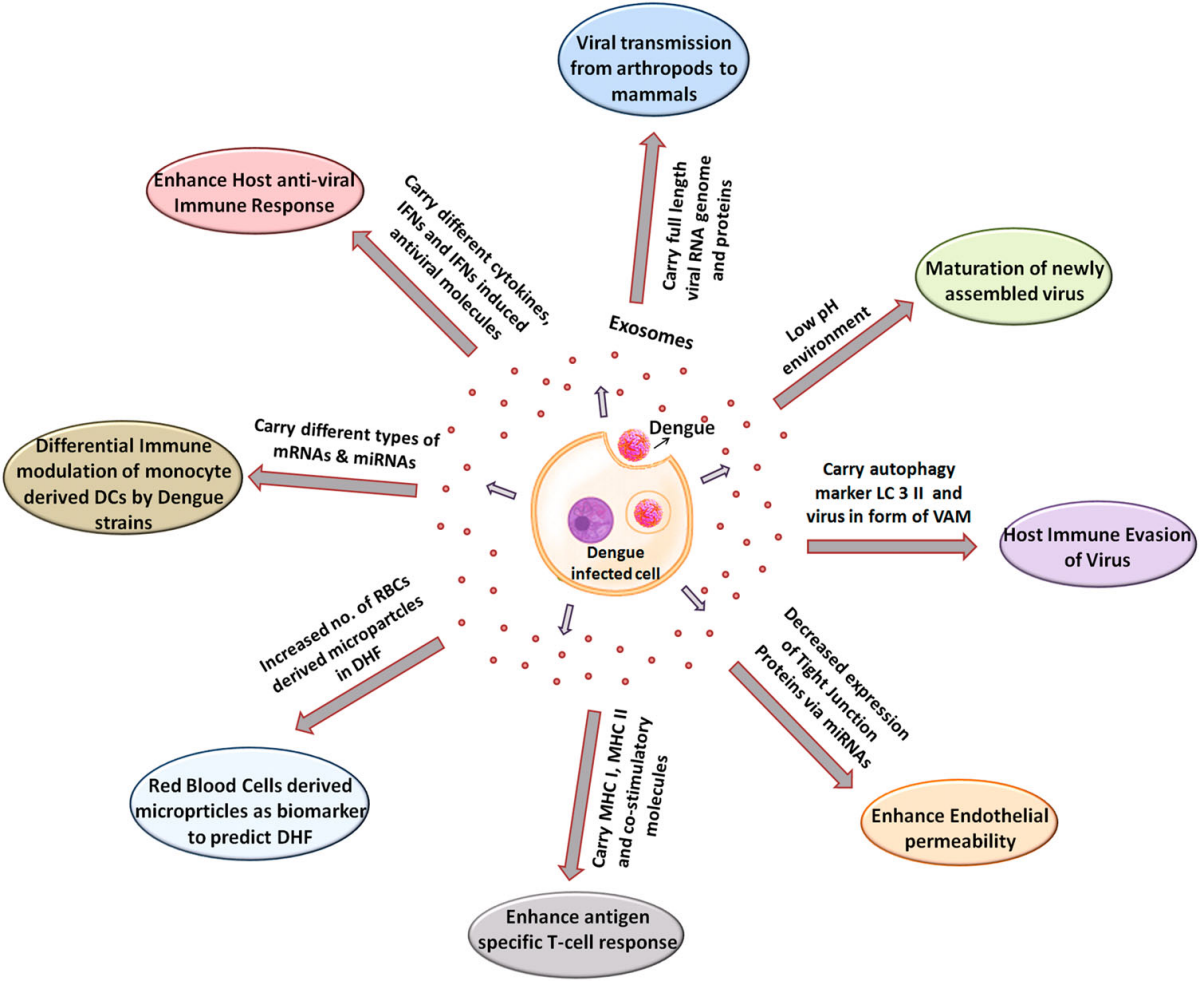
Multidimensional role of exosomes in DENV life cycle. DENV infection modifies the released exosomes/microvesicles content to facilitate its successful life cycle. DENV replication, its particle maturation, packaging, viral transmission from one cell to another, evasion of host immunity and ultimately plethora of pathogenic outcomes depend on the release of microvesicles. Modulation of immunity in different cells such T cells, platelets, RBC and endothelial cells utilize various cargo transported via exosomes. The exosomal cargo contains viral mRNA, viral toxins, host microRNA, transcription factors, mRNAs, receptors. etc.

Here we are going to review all currently available reports on the role of EVs and discuss how they can either contribute positively or negatively for DENV pathogenesis (Figure 1). Figure 1 is depicting the multidimensional roles played by exosomes during the various steps and phases of DENV pathogenesis. The following section of literature will discuss all these facets of DENV pathogenesis that are impacted by exosomal transfer.

### Exosomes helping DENV infection and pathogenesis

In a mosquito gut cell line C6/36 which is very frequently used for DENV replication, DENV2/DENV3-infected cells were reported to secrete EVs [55]. These EVs were carrying full-length DENV2 genome and were infectious to naïve mosquito and mammalian cells like human-skin keratinocytes and blood endothelial cells [55]. This mode of viral transmission was further confirmed via treatment with an exosome-release inhibitor GW4869. GW4869 treatment affected viral burden, interaction of E-protein with exosomal membranes and EV-mediated transmission of viral toxins to naïve human host cells [55]. Exosomes secreted from DENV-infected cells also carry LC3 II, an autophagy marker in addition to viral genome and proteins which protects the virus from anti-dengue neutralizing antibodies, thus enabling the safe viral transmission between the cells [57]. Once inside these microparticles, viruses are reported to acquire altered structural form, called **V**irus of **A**lternative **M**orphology (VAM) [58]. Since exosomes are derived from an endosomal origin, low pH inside exosomal compartment is an important intrinsic feature of these microvesicles. This helps the maturation of newly assembled virus in the exosomal compartment [59].

### DENV and platelet-derived exosomes

**Figure 2.**
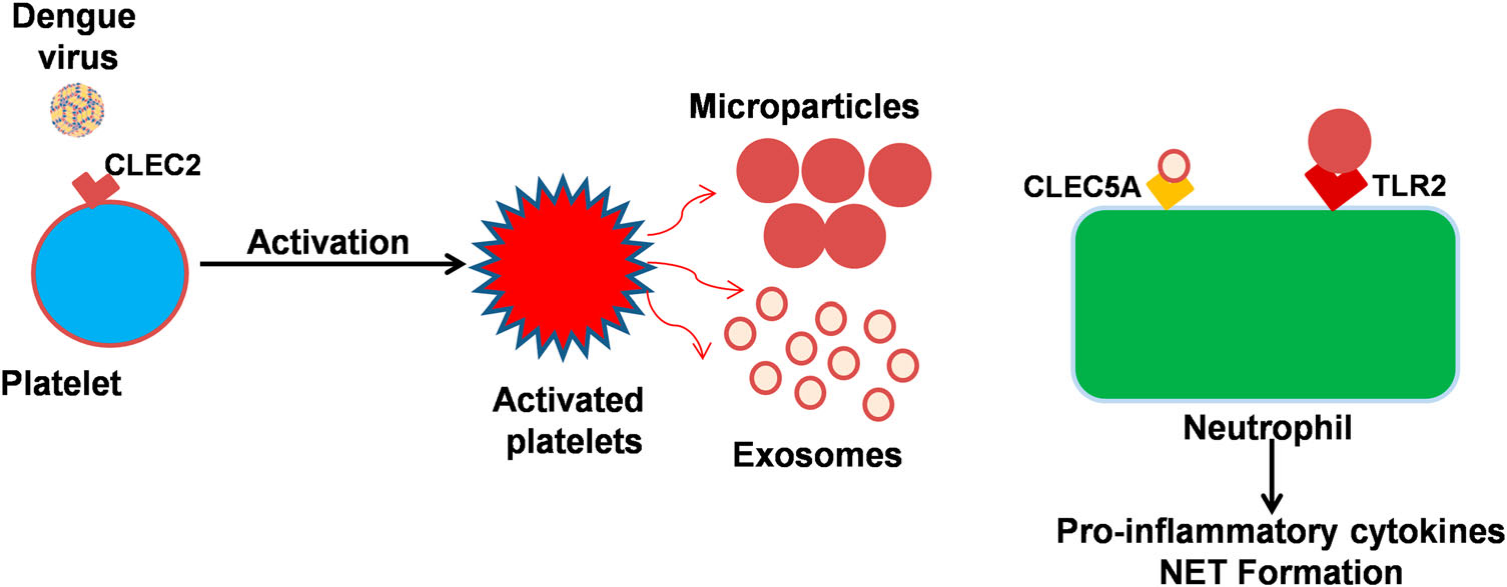
Platelet-derived exosomes and microparticles interact with neutrophils dengue virus binds to platelet surface protein CLEC2 resulting in the release of microparticles and exosomes. The platelet releases microbodies then binds with neutrophil and macrophages resulting in the release of pro-inflammatory cytokines and components of neutrophil extracellular trap (NET) from these cells. Exosomes released from dengue virus-activated platelets also protect the bleeding in virus-infected patients.

Dengue virus (DENV) activates platelets via CLEC2 (C-type lectin like receptor 2) to release EVs which includes both exosomes and microvesicles [60]. DENV-induced release of platelet EVs enhances Neutrophil Extracellular Traps (NETs) formation and causes massive pro-inflammatory cytokine production through activation of CLEC5A and TLR2 on macrophages and neutrophils. Platelet–leukocyte interactions play a very important role in the pathogenesis of infectious diseases specially related with vascular injury, thrombosis, and autoimmunity [61, 62]. A recent study indicated that platelet-derived IL-1β is prominently released in microparticles. This study also concluded that this phenomenon of platelet secretion of IL-1β-rich microparticles is a strong correlate of increased vascular permeability, (schematically represented via cartoon image as Figure 2).

### Exosomes restricting DENV infection and pathogenesis

Exosomes have been shown to deliver IFITM3 (Interferon-induced transmembrane protein 3) from DENV-infected cells to non-infected cells. Exosomes carrying IFITM3 transmit antiviral activities from cell to cell during DENV infection [63]. IFITMs are Interferon-inducible transmembrane proteins 1, 2, and 3 (IFITM1, IFITM2, and IFITM3). They are potent antiviral effector molecules known to suppress the entry of many enveloped viruses and influence the cellular tropism of viruses independent of receptor expression. A cellular model of dengue infection showed that exosomal IFITM3 inhibits the dengue virus entry in both the baseline and interferon-induced conditions [63]. Most importantly, for the first time, this study reported the presence of IFITM inside exosomes in circulation or extracellular environment. This was a remarkable host antiviral strategy conferring viral resistance to cells and tissues located far away from the primary infection site.

Interestingly, another study revealed that IFITM proteins could interfere with the antibody-dependent enhancement (ADE) phenomenon during the secondary DENV infection, which bypassed the IFN-mediated restriction [64]. Chan et.al (2014) showed in human myelogenous leukaemia K562 cells that IFITM proteins can restrict both direct and ADE-associated secondary infection of dengue. K562 cells express FcγRIIa and upon exposure with dengue virus (direct and ADE/secondary infection) appear equally sensitive to IFITM-mediated restriction [63]. This finding, for the first time, connected the phenomenon of ADE with interferon-inducible membrane proteins. This study discussed the possibility whether IFITM proteins could contribute to controlling the secondary infection which might eventually cause DHF. Their experimental data indicated that in human myelogenous leukaemia cell line, IFITM proteins limit the ADE-mediated infection as efficiently as the direct primary infection.

## Role of microRNAs in DENV severity

Various microarray analyses demonstrated aberrant miRNA expression in patient’s plasma as a hallmark of DENV infection. Total plasma samples, endothelial cells, macrophages and dendritic cells, all of them have been reported to display altered microRNA profiling upon DENV infection [65–69]. Endothelial migration response, various inflammatory response and viral replication itself was shown to be regulated via different sets of microRNAs [68, 69]. miR-590-3p, miR-146a, miRNA-3614-5p, miR-24-1-5p, miR-512-5p and miR-4640-3p, miR-125, miR34 and many more have been reported to play a critical role at various stages of DENV pathogenesis [65, 68, 70]. Our previous work has also reported the regulation of VE-cadherin, an adherens junction protein to be regulated by miR-101 upon the exposure of HIV-1 Tat protein [71]. Many recent studies have also identified dysregulated miR-130a and miR-212 expression in colonic epithelium during HIV-1 infection which facilitate epithelial barrier disruption via suppressing the expression of occludin and peroxisome proliferator–activated receptor γ (PPARγ) [72] as well as direct disruption of mucosal epithelial barrier by HIV-1 was also evident [73]. Vascular endothelium is rightly considered as the battlefield of DENV when understanding the hyperpermeability and plasma leakage [74]. Independently if we dissect the microRNA-mediated regulation over endothelial permeability, it suggests that vascular endothelial permeability is, in fact, under robust regulation of microRNA/small RNA. miR-101 mediated regulation of VE-cadherin [71], miR-105-mediated regulation of ZO-1 [47] and miR-155- and miR-126-mediated regulation of PECAM-1 and their impacts on neutrophil rolling and EC junction integrity [75] are just a few examples.

**Figure 3.**
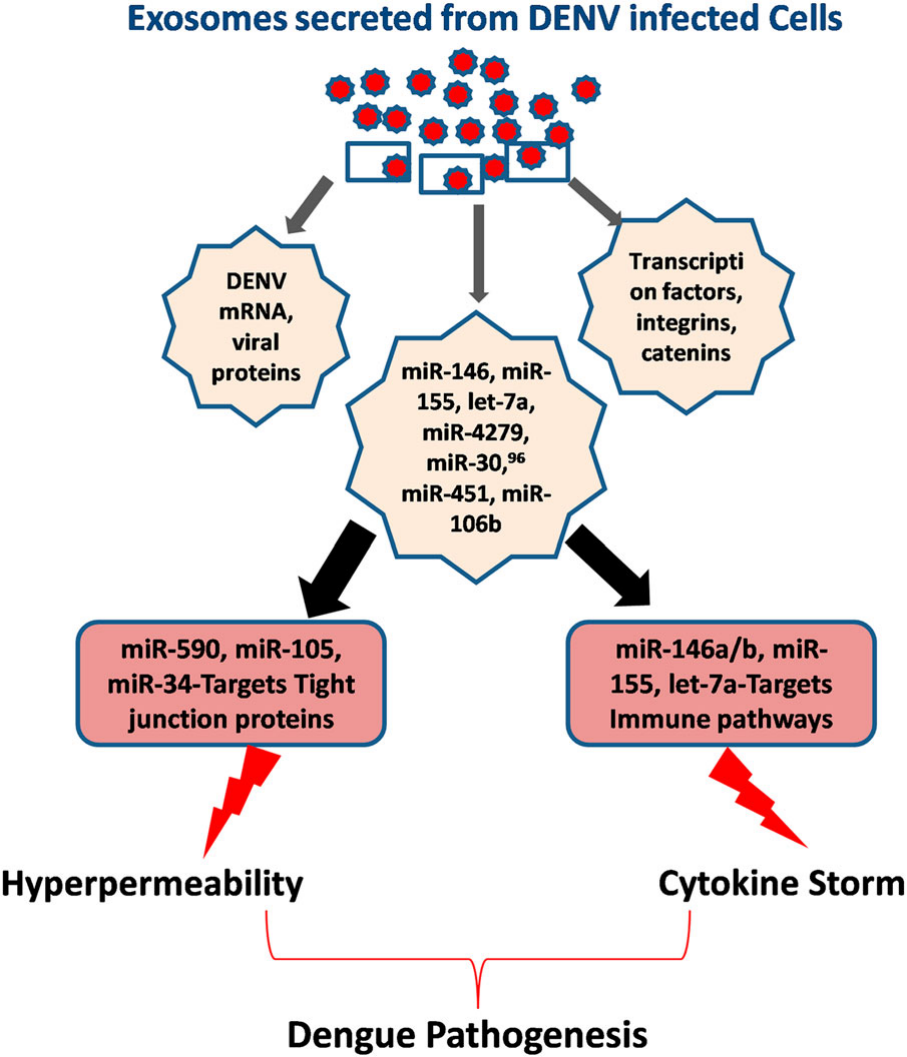
Exosomal microRNA cargo contributes to DENV pathogenesis. Two major aspects of DENV pathogenesis are hyperpermeability and onset of cytokine storm. For bringing out these changes, DENV-infected cells actively modify the exosomal content. They incorporate various microRNAs and transcription factors which causes the above pathological changes in receiving cells and tissues. miR-146, miR-155, let-7a etc are known to modulate innate immune signalling, found to be transported via exosomes. Exosomal miR-105, miR-590, miR-34 and miR-27 etc are known to be targeting the expression of various tight junction proteins which causes the vascular dysfunctions and leads to plasma leakage, a hallmark of dengue haemorrhagic fever.

Apart from endothelial permeability, DENV disease severity is also expressed in the form of hyperactivation or unregulated expression of pro-inflammatory gene networks, a phenomenon is also known as “Cytokine Storm.” Here again microRNAs play a very important role in regulating almost all immune regulatory pathways in various immune cells. miR-146, miR-155, let-7a, miR-4279,miR-30, miR-451, miR-106b, and others have been found to be directly regulating cytokine storm via regulating IL-6/8, CXCL9/10/11, CCL5, TNF-a, IL-10, etc. (Figure 3). This figure explains how two major arms of DENV pathogenesis, i.e. hyperpermeability and cytokine storm are immensely under the influence of microRNA expression profile of the host cells. Previously, our laboratory has also established how the regulation of TRAF3 influences IRF3/IRF7 activation. We reported TRAF3 being regulated by miR-32 in human microglial cells upon the exposure of HIV-1 Tat protein [76]. Our recent finding of Dengue NS5-mediated perturbation of miR-590 and consequent suppression of a de-ubiquitinase protein, USP42 indicated that how microRNAs could play a critical role in DENV pathogenesis [77].

## Exosomes:the competent carrier for microRNA

Several examples for innate immunity modulation by exosomal microRNA are reported so far [78, 79]. Specially in the case of RNA viruses, it has been reviewed extensively elsewhere [49]. The exosomal contents (microRNA, mRNA, viral genes, TF) have been found to be fully functional in distant acceptor cells. Upon entry into recipient cells, microRNAs are shown to downregulate their target gene translation and exosomal long mRNAs were also reported to be translated into functional proteins inside the recipient cells.

A list of exosomal microRNAs targeting endothelial junctional protein, thereby becoming potential contributor for vascular disintegration, is given in Table 1. Preferred loading of microRNA within exosomes and their potent role in the regulation of vascular permeability gives us a new dimension to look for novel pathways to understand the process of DHF.

**Table 1:** . Role of exosomal microRNA in vascular dysfunction.

Exosomal microRNA	Target gene	Functions	References
miR-103	VE-Cadherin, p120-catenin and ZO1	increases vascular permeability	PMID: 29637568
miR-25-3p	KLF2 and KLF4	increases vascular permeability	PMID: 30568162
miR-142-3p	RAB11/FIP2	increases vascular permeability	PMID: 28073833
miR-105	ZO1	increases vascular permeability	PMID: 24735924
miR-210	SMAD4 and STAT6	increases vascular permeability	PMID:29858059
miR-23a	PHD1/PHD2 and ZO1	increases vascular permeability	PMID: 28436951
miR-939	VE-cadherin	increases vascular permeability	PMID: 27693459
miR-200	E-cadherin	increases vascular permeability	PMID: 18381893
miR-138	CTU1, KIAA1274 and RAX	increases vascular permeability	PMID: 29552328

Note: The list of microRNA circulating as exosomal cargo reported to perturb endothelial integrity and affecting vascular functions. This indicates the potentiality of exosomal transport mechanism as a crucial way to impact the vascular health during DENV infection.

Earlier reports showed that exosomes carry lesser amount of small RNA than cellular levels, but interestingly those small RNA fraction were enriched in miRNAs [80]. Another study by Guduric-Fuchs and colleagues also demonstrated that specific subset of miRNAs are selectively loaded into the exosomes, suggesting an active and specific loading mechanism [81].

A recent study demonstrated that microRNAs released in exosomes reflect the cellular microRNA expression profile [82, 83]. It also stated that majority of microRNAs (∼ 66%) are released from source cells passively. miR-16 was identified as a passively released exosomal microRNA and the cellular levels of miR-16 correlated with the their levels found in secreted exosomes [83]. However, a recent report by Squadrito *et.al.* demonstrated that the level of microRNAs loaded in exosomes is determined by the relative levels of the microRNA and their target mRNA inside cell. This is likely to happen if there is an abundance of the target mRNA of certain microRNA, in that case the microRNA will stay bound with target mRNA and this will reduce the loading of microRNA in the exosomes. In the case of higher levels of the microRNA relative to its target mRNA, enhanced loading of the microRNA will be observed in exosomes. This interesting mechanism also suggested that exosomal release pathway is not just to secrete viral factors but also to counter balance the microRNA:mRNA homeostasis in the cells[84].

## Summary and future perspectives

The most staggering aspect of exosomes in DENV pathogenesis is that exosomes or microvesicle-mediated communication can allow the virus to respond or modulate the cellular microenvironment even in the absence of active viral replication. In this review, we have attempted to highlight the notion that a single theory of DHF cannot explain the whole scenario alone. DHF is a phenomenon which occurs at multiple places in the host despite the fact that DENV is not homogenously distributed either temporally or spatially. DENV-infected cells secrete microvesicles loaded with a variety of molecular cargo. Further study of virally modified microvesicles/exosomes will eventually resolve their roles in DENV pathogenesis. An obvious functional overlap between exosome biogenesis and viral budding encourages us to hypothesize that exosomes should be a key influencer for virus life cycle. Exosome research and their precise role in DENV pathogenesis and other viruses are still in infancy. A multidimensional understanding of exosome biology and its significance in viral pathogenesis would certainly give us many useful windows to interfere with viral replication and Pathogenesis.
